# Function of immune cells and effector molecules of the innate immune system in the establishment and maintenance of pregnancy in mammals — A review

**DOI:** 10.5713/ab.24.0257

**Published:** 2024-08-23

**Authors:** Soohyung Lee, Inkyu Yoo, Yugyeong Cheon, Eunhyeok Choi, Seonghyun Kim, Hakhyun Ka

**Affiliations:** 1 Division of Biological Science and Technology, Yonsei University, Wonju, 26493, Korea

**Keywords:** Conceptus, Endometrium, Implantation, Innate Immune System, Pregnancy

## Abstract

In mammalian species, pregnancy is a complex process that involves the maternal recognition of pregnancy, implantation, decidualization, placentation, and parturition. The innate immune system is composed of cellular components, such as natural killer cells, neutrophils, monocytes, and macrophages, and effector molecules, such as cytokines, interferons, antimicrobial peptides, and complement components. The innate immune system plays a critical role as the first line of defense against infection or inflammation to maintain homeostasis and activate the adaptive immunity. During pregnancy, innate immune cells and effector molecules act on the regulation of innate immunity for host defense and processes such as embryo development, implantation, and placentation at the maternal–conceptus interface. In this review, we describe the components of the innate immune system and their functions at the maternal–conceptus interface to establish and maintain pregnancy in animal species that form hemochorial- or epitheliochorial-type placentas, including humans, rodents, ruminants, and pigs.

## INTRODUCTION

The innate immune system is a rapid first-line defense mechanism against infection or inflammation that plays a critical role in preventing infections, maintaining homeostasis, and activating adaptive immunity [1]. The innate immune system is composed of cellular components, including epithelial cells, endothelial cells, natural killer (NK) cells, neutrophils, monocytes, and macrophages, and effector molecules, including cytokines, acute phase proteins (APPs), antimicrobial peptides (AMPs), and complement components [2]. The endometrium is an important site of mucosal immunity in which the innate immune system protects against bacterial and viral infection and signals the presence of pathogens to the acquired immune system [3]. After fertilization, the maternal immune system must tolerate the semi-allogeneic fetus during the implantation period and simultaneously maintain the host defense against potential pathogens for the successful establishment and maintenance of pregnancy [3]. Excessive immune tolerance can cause vulnerability to infection during pregnancy, but inadequate immune tolerance runs the risk of rejecting the fetus and pregnancy failure [4]. Therefore, it is crucial to maintain a balance between immune tolerance toward the semi-allogeneic conceptus and the immune response against potential pathogens. In addition, the cells and effector molecules of the innate immune system play critical roles not only in regulating innate immunity for host defense but also in functions such as embryo development, implantation, and placentation. Thus, it is essential to understand in detail how these cells and effector molecules function at the maternal–conceptus interface to establish and maintain successful pregnancy in humans and domestic animals. This review describes the roles played by innate immune cells and effector molecules in the establishment and maintenance of pregnancy, focusing on animal species that form hemochorial- and epitheliochorial-type placentas.

## IMMUNE CELLS OF THE INNATE IMMUNE SYSTEM

### Neutrophils

Neutrophils are polymorphonuclear innate immune cells characterized by multi-lobulated nuclei and granules and accounting for 50% to 70% of circulating leukocytes [5]. Neutrophils are rapidly recruited to sites of infection following a chemotactic cytokine gradient, and they play a key role in sterile inflammation and infection [5]. Neutrophils have a wide variety of effector functions, including degranulation, phagocytosis, generation of reactive oxygen species (ROS), formation of neutrophil extracellular traps (NETs), participation in tissue repair, and regulation of the adaptive immune system [6]. The granules in neutrophils contain molecule such as AMPs, proteinases, neutrophil elastase, and NETs [5]. NETs contain DNA, histones, and distinct granule proteins that can be used as NET markers [7]. Activated neutrophils induce the activation and maturation of macrophages, dendritic cells, and T cells [7]. After prolonged pathogen contact, neutrophils undergo various cell death mechanisms, including apoptosis, necroptosis, and NET formation [8].

In humans, neutrophils are detected in the decidua during the first trimester of pregnancy, and they increase at the time of labor [6]. The neutrophils in peripheral blood of pregnant women express lower levels of cluster of differentiation (CD) 10 and higher levels of CD15 in the third trimester than the earlier stages of pregnancy [9]. The neutrophils show an increased capacity for phagocytosis, ROS production, and arginase metabolism during pregnancy [10]. Activated neutrophils secrete pro-inflammatory and pro-labor mediators, including cytokines, chemokines, proteases, and prostaglandin E2, which affect physiological processes such as labor, cervical remodeling, uterine contractility, and fetal membrane rupture [5]. In ruminants, interferon (IFN)-τ (IFNT) derived from the conceptus trophectoderm acts as a central signal for maternal recognition of pregnancy, acting in a paracrine manner in the uterus [11]. IFNT induces the expression of IFN-stimulated genes (ISGs) in bovine neutrophils through Janus kinase (JAK) 3 and phosphoinositide 3-kinase (PI3K), which lead to increased production of interleukin (IL)-10 (IL10) and decreased NET formation during maternal recognition of pregnancy [11,12]. In addition, the expression of CD11b, CD31, CD44, and CD62L remains low during pregnancy and increases on the day of abortion or labor [12]. In pigs, the number of neutrophils in the endometrium increases in the uterine lumen several hours after insemination but remains low in endometrial tissue during early pregnancy [13]. Problems such as delayed endometrial apoptosis postpartum can cause persistent inflammation and contribute to pregnancy-associated neutrophilia, and an increase of neutrophils and inflammatory activity can cause infertility or pregnancy loss [11]. Overall, the number of neutrophils in the endometrium during pregnancy increases at mating and then remains low until labor, but any situations harmful to the pregnancy activate neutrophils to transfer signals to other cells locally or systemically. Thus, an appropriate number and activation of neutrophils in the endometrium is important for the establishment and maintenance of pregnancy.

### Natural killer cells

NK cells are generally considered a component of the innate immune system because they lack antigen-specific cell surface receptors and act as major effector cells that control several types of tumors and microbial infections [14]. NK cells have strong cytotoxicity and cytokine-producing effector functions, as well as regulatory functions through their interactions with dendritic cells, macrophages, T cells, and endothelial cells [14]. The activation and effector functions of NK cells depend on the integration of signals from activating and inhibitory receptors [14]. Activating receptors of NK cells include killer cell lectin-like receptor (KLR) K, the natural cytotoxicity receptor family, and CD244 (also known as 2B4); inhibitory receptors include killer cell immunoglobulin (Ig)-like receptor (KIR), two Ig domains and long cytoplasmic tail 1 (KIR2DL1), KIR3DL1, KLRB1, KLRC1, and KLRG1 [15]. Stimulation of the activating receptors induces activating signals that surpass the requisite threshold, resulting in cytokine secretion or direct cellular cytotoxicity [15]. Activation of inhibitory receptors by ligands such as major histocompatibility complex (MHC) class I molecules expressed on the surfaces of normal healthy cells transduces inhibitory signals for self-tolerance to NK cells [16]. The expression of MHC class I is lost in virus-infected cells or tumor cells, which lowers the inhibitory signals. On the other hand, the cellular stress associated with viral infection or tumor development induces the production of ligands that interact with activating receptors of NK cells, causing NK cell–mediated cytotoxicity or cytokine production [16].

Decidual NK (dNK) cells are a specialized type of NK cell found in endometrial decidual tissues during pregnancy, and they have different features from peripheral blood NK cells in humans and rodents [17]. During pregnancy, dNK cells are the most abundant leukocytes, accounting for almost 70% of total decidual leukocytes following recruitment and activation by ovarian hormones in humans [17]. In humans and rodents, dNK cells release cytokines and chemokines to induce trophoblast invasion and spiral artery remodeling for embryo development and placentation [17]. In ruminants, the number of NK cells in the endometrium increases during early pregnancy in cows and sheep [18,19] and the lytic activity of NK cells from peripheral blood mononuclear cells and the endometrium is reduced by serpin family A member 14 purified from uterine flushings in sheep [20]. In pigs, endometrial NK cells are relatively small and agranular lymphocytes, compared with those in rodents and humans, that are transformed into large and granulated forms in the uterus [21]. Porcine endometrial CD16+ NK cells are more abundant in the endometrium than in blood, especially at the conceptus attachment sites, from Day 15 to Day 28 of pregnancy [21]. The cytotoxicity of endometrial NK cells in pigs increases between Day 10 and Day 20 of pregnancy and dramatically decreases on Day 30 [22]. The expression of the *FCGR3A* (CD16) NK cell surface marker is detected in the endometrium of pigs with the highest abundance on Day 15 and decreases thereafter [23]. The recruitment of NK cells into the endometrium is mediated by chemokines such as C-X-C motif chemokine ligand (CXCL) 9, CXCL10, and CXCL11, which are induced by IFN-γ (IFNG) of conceptus origin during early pregnancy [23]. Although the detailed function of endometrial NK cells during pregnancy still needs to be determined, the abundance of NK cells at the maternal–fetal interface and the tight regulation of the expression of activating and inhibitory NK cell receptors in the endometrium suggest that NK cells might play an important role in the establishment and maintenance of pregnancy in mammalian species by modulating the innate immune system.

### Macrophages

Macrophages are derived from circulating monocytes in blood and localized to all tissues in the body [24]. Macrophages play essential roles in innate and adaptive immune responses by detecting, ingesting, and processing foreign materials, dead cells, and other debris, as well as by participating in inflammatory and anti-inflammatory processes [25]. In general, macrophages are classified into two subsets, M1 and M2 macrophages [25]. M1 macrophages are classically activated by IFNG or lipopolysaccharide and produce proinflammatory cytokines such as IL-1β (IL1B), IL12, and tumor necrosis factor-α [26]. M1 macrophages highly express MHC class II molecules, N-formyl peptide receptor 2, G protein-coupled receptor 18, CD38, CD68, CD80, CD86, suppressor of cytokine signaling 3, and nitric oxide synthase [26,27]. M1 macrophages are involved in microbicidal activity, including phagocytosis of pathogens and killing of intracellular bacteria [27]. M2 macrophages are alternatively activated by IL4, IL10, IL13, and transforming growth factor (TGF)-β and involved in immunomodulatory functions, including immune tolerance and resolution of inflammation. They express CD163, CD204, early growth response protein 2, MYC and arginase 1 (ARG1), which are used as M2 markers [26]. M2 macrophages have weak microbicidal activity and secrete the anti-inflammatory cytokine IL10 [27].

Macrophages play an important role in implantation and placentation during pregnancy [28]. Macrophages are rarely present in non-pregnant endometrium, but their number in the decidua increases abundantly during pregnancy through the expression of chemokines [24,28]. In humans, the number of decidual macrophages increases, accounting for 10% to 20% of decidual immune cells during early pregnancy, with the highest levels seen in the first and second trimesters of pregnancy [29,30]. Macrophages are localized around the spiral arteries when trophoblast invasion and spiral artery remodeling are initiated during early pregnancy [24]. Specifically, decidual macrophages express M2 phenotype characteristics, with reduced CD86 expression and increased IL10 and indoleamine 2,3-dioxygenase expression [24]. The number of macrophages increases in the endometrium during the implantation period in ruminants [19,31] and macrophages in the endometrium differentiate into an M2 activation pathway during pregnancy in cows [32]. In pigs, the number of macrophages in the endometrium is higher in sows than in gilts [33]. Macrophages in the endometrium during early pregnancy are localized around blood vessels, express CD163 and ARG1, and produce the anti-inflammatory cytokine IL10 [33,34]. Because endometrial tissue in pigs produces several chemokines CXCL9, CXCL10, and CXCL11 in response to conceptus IFNG signaling during the implantation period [23], it is likely that these chemokines recruit macrophages into the endometrium. Although the proportions of the M1 and M2 phenotypes among endometrial macrophages during pregnancy and their functions are not fully characterized in all species, macrophages might play important roles in the establishment and maintenance of pregnancy by promoting an anti-inflammatory environment and immune tolerance in the endometrium.

## EFFECTOR MOLECULES IN THE INNATE IMMUNE SYSTEM

### Cytokines

Cytokines are a broad group of signaling proteins, such as ILs, IFNs, and chemokines, that induce many physiological processes, including cell growth, migration, inflammation, and immunity [35]. Cytokines are also involved in various reproductive processes, including ovulation, luteolysis, menstruation, implantation, embryo development, placentation, and parturition [35].

### IL1B

IL1B is a potent pro-inflammatory cytokine expressed by hematopoietic cells in response to inflammatory stimuli, and it plays essential roles in the host defense response to infection and injury [36]. IL1B is produced by various types of innate immune cells, such as monocytes and macrophages [36]. IL1B is synthesized as a 31 kDa inactive precursor, pro-IL1B [37]. Full-length pro-IL1B cannot bind to its receptors and needs to be cleaved by caspase-1 prior to secretion in its active form [37]. The receptors of IL1B include IL1 receptor type 1 (IL1R1), IL1R2, IL1R accessory protein (IL1RAP), and a receptor antagonist (IL1RN) [38]. The biological activity of IL1B is enhanced in the presence of soluble IL1R1 and suppressed in the presence of membrane and secreted forms of IL1R2 [38].

In humans, IL1B, IL1R1, and IL1R2 are detected in both the endometrium and the conceptus at the maternal–fetal interface during the implantation period [35]. The endometrial expression of *IL1B* is highest during the late secretory phase and localized to stromal cells, macrophages, uterine NK cells, and endothelial cells [38]. During early pregnancy, IL1B is also detected in villous cytotrophoblasts, syncytiotrophoblasts, the decidua, and macrophages [39]. In mice, *Il1b* is expressed in the uterus, with the highest levels on Days 4 to 5, which corresponds to the time of implantation [40]. In cows, IL1B and IL1R1 are localized in luminal (LE) and glandular epithelial (GE) cells and stromal cells, and the presence of embryos increases endometrial IL1B production [41]. The expression of *IL1B*, *IL1R1*, *IL1RAP*, and *IL1RN* is detected in the endometrium during the estrous cycle and pregnancy in pigs [42]. IL1B is also produced by elongating conceptuses between Days 11 and 12 of pregnancy [42]. Porcine conceptuses express the *IL1B2* gene that is different from the classic *IL1B* gene [42]. The classical *IL1B1* is expressed in the endometrium and macrophages, whereas the embryonic form *IL1B2* is expressed only in the early porcine conceptus during the peri-implantation period [42]. Also in pigs, *IL1R1* and *IL1RAP* are expressed in LE and GE cells of the endometrium, with the highest levels on Day 12 of pregnancy [42]. IL1B plays an important role in the implantation process in several species, including humans, rodents, cows, and pigs, by mediating communication between the conceptus and the endometrium at the maternal–fetal interface [35,39–42]. Although IL1B is well known as a pro-inflammatory cytokine, its roles at the maternal–conceptus interface and in the endometrium of various animal species include the regulation of trophoblast invasion, decidualization, endometrial gene expression, and prostaglandin (PG) production for labor induction [42], suggesting that it might play important roles not only in regulating innate immunity but also in establishing and maintaining pregnancy.

### IL6

IL6 is a multifunctional cytokine with a pleiotropic effect on inflammation, immune response, and hematopoiesis [43]. IL6 is expressed in various cell types, including epithelial cells, fibroblasts, keratinocytes, dendritic cells, lymphocytes, and macrophages [44]. The receptors for IL6 are the IL6 receptor (IL6R) and glycoprotein 130 (GP130), and activation of the IL6 receptor complex by IL6 binding triggers a downstream signaling cascade, such as the JAK-signal transducer and activator of transcription (STAT) signaling pathway [43].

The expression of IL6 and IL6 receptors at the maternal–fetal interface has been found in humans, mice, cow, sheep, and pigs [45–50]. In humans, IL6 is localized to epithelial and stromal cells in the endometrium, and its levels increase during menstruation and the implantation period of pregnancy [46]. In mice, the expression of *Il6* is localized to LE, GE, and stromal cells in the endometrium during the estrous cycle and pregnancy [47]. *Il6*-deficient mice show increased fetal resorption and delayed parturition [45]. The expression of *IL6* in the endometrium decreases during early pregnancy compared to cyclic heifers [48], while the expression of *IL6* in the endometrium increases in pregnant sheep compared to non-pregnant sheep [49]. IL6 and IL6 receptors are also expressed in the endometrium during pregnancy in pigs [50]. The expression of *IL6*, *IL6R*, and *GP130* increases during mid- to late pregnancy and is localized to epithelial and stromal cells in the endometrium [50]. IL6 is involved in embryo implantation, placental development, and immune tolerance at the maternal–fetal interface in humans, rodents, and pigs [45].

### IL10

IL10 is a key anti-inflammatory cytokine that protects the host from an excessive immune response to infection and inflammation [51]. IL10 is produced by various cell types, such as dendritic cells, macrophages, neutrophils, T helper 2 (Th2) cells, and some epithelial cells [51]. The receptors for IL10 are a heterotetramer consisting of two IL10 receptor-α (IL10RA) subunits and two IL10 receptor-β (IL10RB) subunits [51]. The complex of IL10 and its receptors engages the JAK-STAT signaling pathway and inhibits the nuclear factor (NF)-κB signaling pathway [51].

The expression of IL10 and IL10 receptors has been observed at the maternal–conceptus interface in humans, mice, ruminants, and pigs [34,49,52,53]. In humans and mice, IL10 is produced in trophoblast cells, decidual cells, and decidual immune cells, including dendritic cells, macrophages, dNK cells, and regulatory T cells [54]. In humans, the expression of IL10 and IL10 receptors increases in the decidua during early pregnancy, compared with the menstrual cycle [55]. In cows, the expression of *IL10* in the endometrium increases during early pregnancy compared to non-pregnant animals and IFNT induces the production of IL10 in endometrial epithelial cells [48]. In sheep, the expression of *IL10* in the endometrium is higher during mid-pregnancy than early pregnancy and the cyclic stage [49]. In pigs, the expression of *IL10* increases on Day 15 of pregnancy and is localized to LE and stromal cells and macrophages in the endometrium [34]. The IL10RA protein is localized to LE, endothelial, stromal, and T cells, and *IL10RB* mRNA is localized to LE cells in the endometrium. The endometrial expression of IL10 receptors is induced by estrogen, IL1B, and/or IFNG in pigs [34]. In humans, decreased levels of IL10 are associated with adverse pregnancy outcomes, including abnormal placentation, recurrent spontaneous abortion, preterm birth, and preeclampsia [54]. Thus, IL10 plays a crucial role in the establishment and maintenance of pregnancy in various animal species by regulating maternal immune activation at the maternal–fetal interface.

### IL15

IL15 is a pleiotropic cytokine that affects the development, maintenance, and function of T cells, NK cells, natural killer T cells, and dendritic cells [56]. IL15 is produced by various cell types, including monocytes, macrophages, dendritic cells, fibroblasts, and epithelial cells [56]. The receptor for IL15 consists of three subunits, IL15 receptor-α (IL15RA), IL2 receptor-β (IL2RB), and IL2R-γ (IL2RG) [56]. The interaction between IL15 and its receptors engages JAK-STAT signaling, leading to the proliferation, differentiation, and activation of T cells and NK cells [57,58]. Additionally, it provides protection from apoptosis in many cell types, including immune cells such as B cells, monocytes, and macrophages, and non-immune cells such as epithelial cells, keratinocytes, and hepatocytes [57].

IL15 is expressed at the maternal–fetal interface and associated with successful and healthy pregnancy in humans, mice, and cows [59–62]. In humans, the IL15 protein is localized in GE, stromal, and vascular endothelial cells in the endometrium during the menstrual cycle [59]. In both mice and humans, IL15 produced by stromal cells, decidual cells, and macrophages induces the differentiation of uterine NK cells and promotes decidualization during pregnancy [59,60]. In humans, IL15 is expressed in endometrial stromal cells, promotes proliferation and invasion of endometrial stromal cells, and decreases activation of NK cells by downregulating the expression of IFNG, granzyme B, and activating receptors, NCR2 and KLRK1, in NK cell [61]. In cows, IL15 is expressed during early pregnancy in the endometrium, but its expression is not affected by conceptus implantation and placentation [62]. In humans and mice, dysregulation of IL15 expression is associated with adverse pregnancy outcomes, including fetal growth restriction, abnormal decidual formation, impaired spiral artery remodeling, recurrent miscarriage, and preeclampsia [63]. Therefore, IL15 and its receptors might play important roles in establishing and maintaining pregnancy in various animal species by regulating the function of endometrial cells and immune cells.

### IFNs

IFNs are cytokines with powerful antiviral activity that influence both the innate and adaptive immune systems [64]. They are secreted from host cells in response to a variety of stimuli, including viral infection [64]. IFNs are divided into three types, type I, type II, and type III, based on their sensitivity to pH, amino acid sequence homology, crystal structure, and functional properties [64]. The interaction between IFNs and their receptors directs a variety of biological functions through signal transduction pathways, including the JAK-STAT, mitogen-activated protein kinase, PI3K-Akt, and NF-κB pathways [64,65]. Type I IFNs, including IFN-α (IFNA), IFN-β, IFN-δ (IFND), and IFNT, exhibit a wide variety of biological functions, including the suppression of viral replication, the induction of apoptosis in infected cells, and the stimulation of cytotoxic activity in NK cells and cytotoxic T cells [64,66]. Interestingly, in ruminants, IFNT derived from the conceptus trophectoderm during early pregnancy acts as a maternal recognition of pregnancy signal [67]. IFNG, the only type II IFN, mediates various biological functions, including the stimulation of antigen presentation, upregulation of pathogen recognition, increase of MHC class I and II expression, enhancement of antimicrobial activity, and activation of M1 proinflammatory macrophages [68,69]. Type III IFNs, including IFNL, are also associated with protection against viral infections, the expression of MHC molecules, T cell differentiation, immunomodulation, and autoimmunity [70].

IFNs are produced at the maternal–fetal interface in humans, rodents, ruminants, and pigs [71–73]. In humans, IFNs, including IFNA, IFNE, and IFNG, are expressed in the endometrium and/or trophoblast cells during early pregnancy [74–76]. These IFNs are associated with endometrial gene expression and antiviral response [77]. In mice, IFNA and IFNG are expressed by the decidua, trophoblast cells, and uterine NK cells [72]. IFNs are involved in embryo implantation, spiral artery development, and decidualization in mice [10,77]. In ruminants, such as sheep, goats, and cows, IFNT is expressed by the trophectoderm of the early-stage conceptus and acts as a signal for maternal recognition of pregnancy by preventing luteolysis [71]. IFNT also stimulates the expression of ISGs and many other genes, including those involved in cell proliferation, migration, attachment, transport of glucose and amino acids, and production of proteases and their inhibitors [71,78]. In pigs, elongating conceptuses secrete IFND and IFNG during the peri-implantation period [79]. Unlike IFNT in ruminants, IFND and IFNG are not involved in the anti-luteolytic effect in pigs [79]. IFNG induces the expression of many endometrial genes, including ISGs, MHC class I and II molecules, chemokines and chemokine receptors, and matrix metalloproteinase [73,79,80]. Thus, IFNs play critical but species-specific roles in the establishment and maintenance of pregnancy by inducing many endometrial genes, regulating the immune response and immune tolerance, and/or acting as a signal for maternal recognition of pregnancy.

### Chemokines

Chemokines, a group of small chemoattractant cytokines with a molecular weight of 8 to 10 kDa, are classified into four subfamilies: the cysteine (C), cysteine-cysteine (CC), cysteine-X-cysteine (CXC), and cysteine-X-X-X-cysteine (CX3C) motifs [81]. There are two families of chemokine receptors, conventional and atypical chemokine receptors (ACKRs) [81]. Conventional chemokine receptors are G-protein coupled receptors that typically activate intracellular signaling pathways by chemokine binding, whereas ACKRs, which structurally resemble conventional chemokine receptors, cannot transduce intracellular signals [81]. Chemokines and chemokine receptors are expressed in various cell types, including dendritic cells, neutrophils, monocytes, lymphocytes, endothelial cells, and epithelial cells, and they are involved in host defense as well as cell functions such as proliferation, differentiation, migration, apoptosis, and angiogenesis [81].

In humans, chemokines are highly expressed in the decidua, decidual stromal cells, and primary trophoblasts, and chemokine receptors are expressed in trophoblasts, decidual stromal cells, NK cells, T cells, and macrophages [82]. Chemokines secreted by decidual cells are involved in recruiting NK cells, T cells, and monocytes into the decidua during early pregnancy in humans [82]. Chemokines also affect placentation by inducing the proliferation, invasion, and differentiation of extravillous cytotrophoblast cells in humans [82]. In mice, chemokines promote uterine endometrial receptivity and angiogenesis [83]. In goats and sheep, chemokines induce the migration and adhesion of trophoblast cells [84,85]. In cows, chemokines and chemokine receptors are expressed in the endometrium during the estrous cycle and pregnancy, and the endometrial expression of chemokines is higher during early pregnancy than at other times [86]. IFNT secreted by implanting bovine conceptuses increases the expression of chemokines, including CCL8 and CXCL10 [86]. In pigs, chemokines and chemokine receptors are detected at the maternal–conceptus interface [23,87]. Conceptus-derived IFNG increases the expression of chemokines in the endometrium on Day 15 of pregnancy in pigs [73]. Chemokines induce the recruitment of immune cells and the proliferation and migration of trophectoderm cells at the maternal–conceptus interface in pigs [23,87]. In addition, an interaction between CXCL12 and CXCR4 is involved in the Th1/Th2 balance at the maternal–conceptus interface during early pregnancy in humans and mice [83]. The abnormal expression of chemokines or chemokine receptors causes various pregnancy complications, such as preeclampsia, recurrent spontaneous abortion, preterm birth, and pregnancy loss in humans and rodents [82]. Overall, chemokines produced by endometrial epithelial, stromal, and endothelial cells and subsets of leukocytes in the endometrium in humans, mice, ruminants, and pigs play important roles in regulating the proliferation and migration of trophoblast cells, recruiting immune cells into the endometrium, and activating the immune system during early pregnancy.

### Acute phase proteins

Acute phase proteins, including C-reactive protein (CRP), fibrinogen, haptoglobin, lipocalin-2, and serum amyloid As (SAAs), are plasma proteins that rapidly increase in blood levels, by 5- to 1,000-fold, in response to inflammation, infection, and trauma [88]. APPs are induced by proinflammatory cytokines and produced mainly by hepatocytes, but local inflammatory cells, including neutrophils, macrophages, and epithelial cells, in the skin, gut, and lung can also produce them [88]. The expression levels of APPs remain high during chronic infection and chronic inflammatory disease, and an increase in circulating APPs in the blood is thought to be beneficial to the host organism by inhibiting microbial growth and restoring homeostasis [88]. The levels of APPs increase dramatically during inflammatory diseases, and they are considered to be sensitive markers of inflammation [89]. APPs are also involved in many reproductive processes, including pregnancy.

In humans, SAAs are synthesized locally in the endometrium, fetal membranes, and placenta [90]. Plasma levels of SAAs increase during both normal pregnancy and abnormal pregnancy, such as premature rupture of membranes, preeclampsia, gestational diabetes, and recurrent spontaneous abortion [90]. CRP levels in serum are also higher during pregnancy than at other times in humans [91]. In mice, the expression of lipocalin-2 increases dramatically after parturition [92]. In cows, the expression of *SAA3* and *haptoglobin* is high in the endometrium during late postpartum compared to early postpartum [93], and plasma concentrations of SAA, haptaglobin, and fibrinogen are higher during pregnancy than during the cyclic stage in sheep [94]. In pigs, *SAA3* and *haptoglobin* are expressed at the maternal–conceptus interface during the estrous cycle and pregnancy [95,96]. The expression of *SAA3* in the endometrium is highest at the time of conceptus implantation and increased by IL1B and IFNG secreted by the implanting conceptuses during early pregnancy [95]. SAAs are considered to promote the development, differentiation, and invasion of the trophoblast in humans [97], and haptoglobin promotes the development of porcine embryos *in vitro* [96]. APPs are also associated with reproductive diseases, including ovarian cancer, endometritis, premature rupture of membranes, preeclampsia, gestational diabetes, and recurrent spontaneous abortion in humans, mice, horses, goats, and pigs [89,90]. These data suggest that APPs play important roles not only in regulating innate immunity but also in a variety of reproductive processes during the establishment and maintenance of pregnancy.

### Antimicrobial peptides

AMPs, also called host defense peptides, are small peptides that play important roles in the innate immune system of various organisms, including microorganisms, amphibians, and mammals [98]. AMPs are classified based on their source, activity, and structural characteristics and include cathelicidins, defensins, S100A calcium-binding proteins (S100As), peptidase inhibitor 3 (PI3), and secretory leukocyte protease inhibitor (SLPI) [98]. AMPs have a wide range of inhibitory effects against bacteria, fungi, parasites, and viruses and act as natural antibiotics that protect mammalian hosts from microbes [98]. The direct antimicrobial mechanisms of AMPs are mediated by membrane translocation and disruption [99]. AMPs also have immunomodulatory properties through cell signaling, activation of immune cells, regulation of pro-inflammatory and anti-inflammatory responses, and recruitment of immune cells [98]. In addition, AMPs such as S100As have unique intracellular functions, such as cellular development, regulation of cytoskeletal organization, transcription, tissue repair, and homeostasis [100].

Most AMPs are produced by epithelial and inflammatory cells in various tissues, including in the lungs, skin, guts, and female reproductive tract, and AMP deficiency causes vulnerability to infection and inflammation [101]. During pregnancy, AMPs such as defensins, cathelicidins, S100As, PI3, and SLPI are expressed in the female reproductive tract in many species, including humans, mice, cows, and pigs [102–108]. In humans, inappropriate AMP expression and regulation are associated with various adverse pregnancy outcomes, including eclampsia, retarded fetal growth, recurrent miscarriage, and premature rupture of membranes [101]. These indicate that AMPs expressed in the female reproductive tract during pregnancy in various species appear to play important roles in maintaining innate immunity to protect the maternal–conceptus interface from microbial infection and successfully establish and maintain pregnancy in mammals.

### Complement system

The complement system is a major part of the innate immune system, serving as a surveillance system against pathogens and altered host cells [109]. The complement system consists of soluble components produced by the liver, which circulate in plasma, and by local extrahepatic tissues [109]. The complement system is activated by three distinct pathways: the classical, lectin, and alternative pathways. These pathways initiate the complement cascade, which is amplified through the sequential cleavage of complement components by enzymatic reactions [109]. The activation of these three pathways results in the cleavage of inactive C3 protein, the central component of the complement system, into its functional C3a and C3b fragments [109]. As a consequence of the complement cascade, those fragments of complement components induce various functions, including direct lysis of pathogens, enhanced opsonization, recruitment of immune cells, activation of endothelial cells, and the regulation of adaptive immunity [110]. To prevent overstimulation, which could be detrimental to host cells at sites of infection, complement activation is tightly regulated by complement control proteins such as membrane cofactor protein (CD46), decay accelerating factor (CD55), membrane attack complex inhibitory protein (protectin or CD59), and complement receptor 1–related gene/protein y (Crry) [111]. The dysregulation of complement activation is associated with increased susceptibility to infections and non-infectious diseases such as autoimmunity, chronic inflammation, thrombotic microangiopathy, graft rejection, and cancer [111].

The complement system is expressed at the maternal–fetal interface, and its inappropriate activation causes adverse pregnancy outcomes such as implantation failure, miscarriage, preeclampsia, and preterm birth in humans and rodents [112]. In humans, complement components, including C1q, C3, and C4, are expressed in endometrial cells and trophoblast cells [113,114], and complement control proteins such as *CD46*, *CD55*, and *CD59* are expressed in the endometrium [115]. In mice, *C3* is expressed in endometrial epithelial cells [116]. Crry, a murine homologue to human CD46 and CD55, has important roles in conceptus survival, blood pressure regulation, and fetal growth during pregnancy in mice [117]. In humans and rats, inactivated C3b (iC3b) promotes embryonic growth and placental development [112]. In mice, the interaction between iC3b and complement receptor 3 affects the production of IL10 and TGF-β1, anti-inflammatory cytokines, in the decidua and placenta during the late stage of pregnancy [118]. In cows, the expression of complement components and complement control proteins increases in the endometrium during the pre-implantation period compared to non-pregnant cows and modulate the maternal immune system [119]. In pigs, complement components and complement control proteins are expressed at the maternal–conceptus interface during the estrous cycle and pregnancy, and conceptus-derived IFNG increases the expression of complement components and complement control protein during early pregnancy (Lee and Ka, unpublished data). Overall, the complement system might play important roles not only in protecting the maternal–fetal interface from microbial infection but also in the growth and development of conceptuses and the placenta during pregnancy.

## CONCLUSION

**Figure 1:**
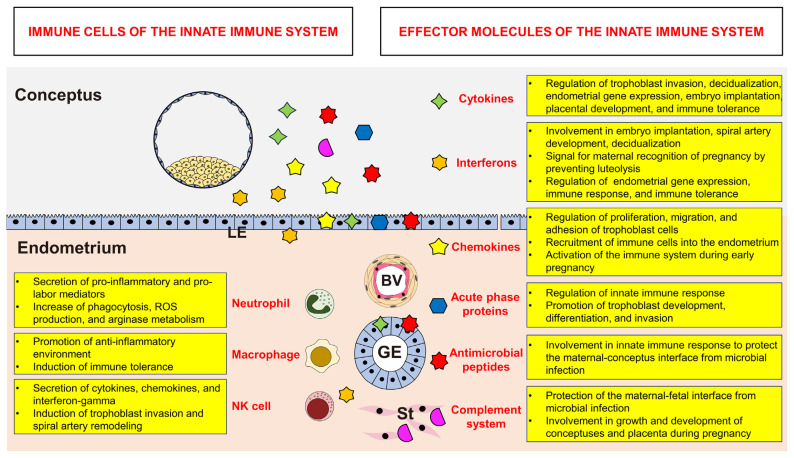
Overview of the roles played by immune cells and effector molecules from the innate immune system at the maternal–conceptus interface during pregnancy in animal species.

During pregnancy, the maternal endometrium experiences a unique and paradoxical immunological situation. The maternal immune system must tolerate the semi-allogeneic fetus during the implantation period and simultaneously maintain its host defense against potential pathogens to ensure the successful establishment and maintenance of pregnancy [3]. Increased immune activation during pregnancy raises the risk of fetal rejection and pregnancy failure, but inadequate maternal immune activation can lead to vulnerability to infections at the maternal–fetal interface [4]. This review has summarized the types of cells and effector molecules found in the innate immune system and their roles at the maternal–fetal interface to establish and maintain pregnancy in some animal species (Figure 1). During pregnancy, neutrophils, NK cells, and macrophages are recruited to the endometrium. Those immune cells, other endometrial cells, and the conceptus produce cytokines, interferons, chemokines, AMPs, and complements. Those effector molecules influence the maternal immune system to both develop tolerance to the semi-allogeneic conceptus and maintain the host defense against potential pathogens that could harm the mother and lead to pregnancy complications. In addition, effector molecules derived from the endometrium and the conceptus are involved not only in mediating the innate immune responses of the endometrium but also in regulating the proliferation, differentiation, and function of trophoblast and endometrial cells to enable successful implantation, placentation, and fetal development. Although many studies have examined the roles of immune cells and effector molecules from the innate immune system during pregnancy, further studies are still needed to elucidate the detailed mechanisms of these cells and effector molecules at the maternal–conceptus interface. Understanding the function of the innate immune system will enable future research to develop strategies for preventing adverse pregnancy outcomes and maintaining fertility in many mammalian species.
